# SARS-CoV-2 brainstem encephalitis in human inherited DBR1 deficiency

**DOI:** 10.1084/jem.20231725

**Published:** 2024-07-18

**Authors:** Yi-Hao Chan, Vanja Lundberg, Jérémie Le Pen, Jiayi Yuan, Danyel Lee, Francesca Pinci, Stefano Volpi, Koji Nakajima, Vincent Bondet, Sanna Åkesson, Noopur V. Khobrekar, Aaron Bodansky, Likun Du, Tina Melander, Alice-Andrée Mariaggi, Yoann Seeleuthner, Tariq Shikh Saleh, Debanjana Chakravarty, Per Marits, Kerry Dobbs, Sofie Vonlanthen, Viktoria Hennings, Karolina Thörn, Darawan Rinchai, Lucy Bizien, Matthieu Chaldebas, Ali Sobh, Tayfun Özçelik, Sevgi Keles, Suzan A. AlKhater, Carolina Prando, Isabelle Meyts, COVID Human Genetic Effort, Paul Bastard, Alessandro Borghesi, Aziz Bousfiha, Oksana Boyarchuk, Petter Brodin, Jacinta Bustamante, Giorgio Casari, Rémi Chevalier, John Christodoulou, Roger Colobran, Antonio Condino-Neto, Juan Carlos Aldave Becerra, Lisa Arkin, Evangelos Andreakos, Christian W. Thorball, Sara Espinosa, Carlos Flores, Amyrath Geraldo, Rabih Halwani, Nevin Hatipoğlu, Brahim Melaiki, Jacques Fellay, Alenka Gagro, Yuval Itan, Chandima Jeewandara, Filomeen Haerynck, Davood Mansouri, Leslie Naesens, Lisa F.P. Ng, Keisuke Okamoto, Pere Soler-Palacin, Laurent Renia, Aurora Pujol Onofre, Igor B. Resnick, José Luis Franco Restrepo, Jacques G. Rivière, Anna Scherbina, Anna Šedivá, Mikko R.J. Seppänen, Helen Su, Stuart G Tangye, Sehime G. Temel, Ahmad Abou Tayoun, Stuart Turvey, K.M. Furkan Uddin, Diederik van de Beek, Tom Le Voyer, Donald C. Vinh, Shen-Ying Zhang, Jean-Laurent Casanova, Michael R. Wilson, Jérémie Rosain, Emmanuelle Jouanguy, Mélodie Aubart, Laurent Abel, Trine H. Mogensen, Qiang Pan-Hammarström, Daxing Gao, Darragh Duffy, Aurélie Cobat, Stefan Berg, Luigi D. Notarangelo, Oliver Harschnitz, Charles M. Rice, Lorenz Studer, Jean-Laurent Casanova, Olov Ekwall, Shen-Ying Zhang

**Affiliations:** 1 St. Giles Laboratory of Human Genetics of Infectious Diseases, Rockefeller Branch, The Rockefeller University, New York, NY, USA; 2 Department of Pediatrics, Institute of Clinical Sciences, The Sahlgrenska Academy at the University of Gothenburg, Gothenburg, Sweden; 3 Department of Rheumatology and Inflammation Research, Institute of Medicine, The Sahlgrenska Academy at the University of Gothenburg, Gothenburg, Sweden; 4 Laboratory of Virology and Infectious Disease, The Rockefeller University, New York, NY, USA; 5 The Center for Stem Cell Biology, Sloan Kettering Institute for Cancer Research, New York, NY, USA; 6 Laboratory of Human Genetics of Infectious Diseases, Necker Branch, INSERM, Paris, France; 7 Paris City University, Imagine Institute, Paris, France; 8 Human Technopole, Viale Rita Levi-Montalcini, Milan, Italy; 9 Rheumatology and Autoinflammatory Diseases, IRCCS Giannina Gaslini Institute, Genoa, Italy; 10 Department of Neuroscience, Rehabilitation, Ophthalmology, Genetics, Maternal and Child Health, University of Genoa, Genoa, Italy; 11 Translational Immunology Unit, Institut Pasteur, Paris City University, Paris, France; 12 Department of Pediatrics, Division of Critical Care, University of California San Francisco, San Francisco, CA, USA; 13 Department of Medical Biochemistry and Biophysics, Division of Immunology, Karolinska Institutet, Stockholm, Sweden; 14 Department of Pediatrics, Härnösand Hospital, Region Västernorrland, Sundsvall, Sweden; 15 Laboratory of Virology, Assistance Publique-Hôpitaux de Paris (AP-HP), Cochin Hospital, Paris, France; 16 Department of Pediatric Dentistry, Sundsvall, Region Västernorrland, Sundsvall, Sweden; 17 Weill Institute for Neurosciences, University of California, San Francisco, San Francisco, CA, USA; 18 Department of Neurology, University of California, San Francisco, San Francisco, CA, USA; 19 Department of Medicine, Huddinge, Hematology Unit, Therapeutic Immunology and Transfusion, Karolinska University Hospital, Stockholm, Sweden; 20 Department of Clinical Science, Intervention and Technology, Karolinska Institutet, Stockholm, Sweden; 21 Division of Intramural Research, Laboratory of Clinical Immunology and Microbiology, National Institute of Allergy and Infectious Diseases, National Institutes of Health, Bethesda, MD, USA; 22 Department of Pediatrics, Mansoura University Children’s Hospital, Faculty of Medicine, Mansoura University, Mansoura, Egypt; 23 Department of Molecular Biology and Genetics, Bilkent University, Ankara, Turkey; 24 Necmettin Erbakan University, Konya, Turkey; 25 College of Medicine, Imam Abdulrahman Bin Faisal University, Dammam, Saudi Arabia; 26 Department of Pediatrics, King Fahad University Hospital, Al-Khobar, Saudi Arabia; 27 Faculty of Pequeno Príncipe, Pesquisa Pelé Pequeno Príncipe Institute, Curitiba, Brazil; 28 Department of Pediatrics, University Hospitals Leuven, Laboratory for Inborn Errors of Immunity, KU Leuven, Leuven, Belgium; 29 Department of Pediatric Neurology, Necker-Enfants Malades Hospital, AP-HP, Paris, France; 30 Department of Biomedicine, Aarhus University, Aarhus, Denmark; 31 Division of Life Science and Medicine, Department of General Surgery, The First Affiliated Hospital of USTC, University of Science and Technology of China, Hefei, China; 32 Division of Life Sciences and Medicine, Institute of Immunology and the CAS Key Laboratory of Innate Immunity and Chronic Disease, University of Science and Technology of China, Hefei, China; 33 Department of Pediatrics, Necker Hospital for Sick Children, AP-HP, Paris, France; 34 Howard Hughes Medical Institute, New York, NY, USA

## Abstract

Inherited deficiency of the RNA lariat–debranching enzyme 1 (DBR1) is a rare etiology of brainstem viral encephalitis. The cellular basis of disease and the range of viral predisposition are unclear. We report inherited DBR1 deficiency in a 14-year-old boy who suffered from isolated SARS-CoV-2 brainstem encephalitis. The patient is homozygous for a previously reported hypomorphic and pathogenic DBR1 variant (I120T). Consistently, *DBR1* I120T/I120T fibroblasts from affected individuals from this and another unrelated kindred have similarly low levels of DBR1 protein and high levels of RNA lariats. *DBR1* I120T/I120T human pluripotent stem cell (hPSC)–derived hindbrain neurons are highly susceptible to SARS-CoV-2 infection. Exogenous WT *DBR1* expression in *DBR1* I120T/I120T fibroblasts and hindbrain neurons rescued the RNA lariat accumulation phenotype. Moreover, expression of exogenous RNA lariats, mimicking DBR1 deficiency, increased the susceptibility of WT hindbrain neurons to SARS-CoV-2 infection. Inborn errors of DBR1 impair hindbrain neuron–intrinsic antiviral immunity, predisposing to viral infections of the brainstem, including that by SARS-CoV-2.

## Teaser

Chan et al. report inherited DBR1 deficiency as a genetic etiology of isolated SARS-CoV-2 brainstem encephalitis. DBR1 I120T/I120T patient-specific hPSC-derived hindbrain neurons display enhanced accumulation of RNA lariats resulting in increased susceptibility to SARS-CoV-2 infection, thereby underlying SARS-CoV-2 brainstem encephalitis.

## Introduction

The first cases of SARS-CoV-2 encephalitis were described in March 2020, about 5 mo after the start of the COVID-19 pandemic (Moriguchi et al., 2020; Ye et al., 2020). It progressively became clear that encephalitis following SARS-CoV-2 infection comprises several different types of encephalopathy, probably due to different mechanisms of disease (Aubart et al., 2022; Cho et al., 2023; Ellul et al., 2020; Siow et al., 2021). These SARS-CoV-2–related forms of encephalitis have an estimated overall prevalence of about 2/10,000. In most cases, encephalitis occurs weeks or months after the acute respiratory viral infection and presents as post-infectious autoimmune encephalitis (Holroyd and Conway, 2023). However, encephalitis may also occur in severe cases of COVID-19 pneumonia or multisystem inflammatory syndrome in children (MIS-C) with the inflammation of multiple organs, including the brain (Olivotto et al., 2021). No particular distribution by age, sex, or ancestry has been observed in either of these types of SARS-CoV-2–related encephalitis, and overall mortality for these two forms is about 13%. Isolated encephalitis, which defines a third class of SARS-CoV-2–related encephalopathy, is extremely severe and has been reported only rarely (Cho et al., 2023; Ellul et al., 2020; Moriguchi et al., 2020; Siow et al., 2021; Ye et al., 2020). Acute SARS-CoV-2 encephalitis often involves brainstem lesions, as revealed by brain imaging data, but lesions in other parts of the brain have also been reported. Over the last 3 years, we and others have shown that critical COVID-19 pneumonia results from inborn errors of, or autoantibodies against, type I interferons (IFNs) in about 15% of cases (Bastard et al., 2021a; Casanova and Abel, 2021, 2022; Zhang et al., 2022). We also recently discovered recessive deficiencies of the OAS-RNase L pathway resulting in unchecked inflammatory responses to SARS-CoV-2 in mononuclear phagocytes, underlying MIS-C in about 1% of the patients studied (Lee et al., 2023). The pathogenesis of SARS-CoV-2 encephalitis remains much less clear. Based on our previous studies of other types of viral encephalitis, including herpes simplex virus 1 encephalitis in particular (Zhang et al., 2021), we hypothesized that inborn errors of brain-intrinsic immunity might underlie isolated SARS-CoV-2 encephalitis. We tested this hypothesis by performing whole-exome/genome sequencing for a cohort of 16 patients from the COVID Human Genetic Effort (https://www.covidhge.com) cohort who developed isolated encephalitis during acute SARS-CoV-2 infection.

## Results and discussion

**Figure 1.**
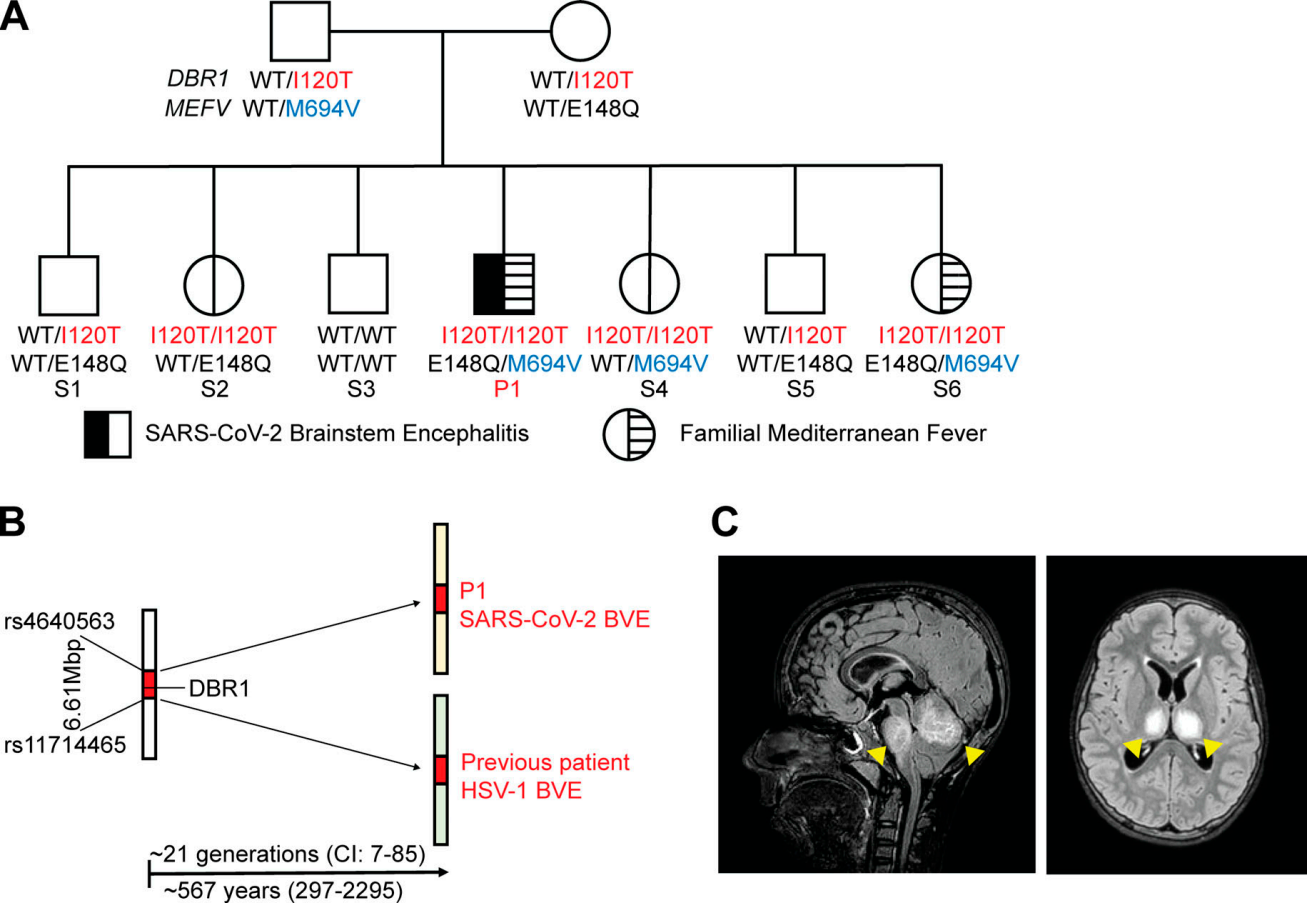
**A patient with SARS-CoV-2 brainstem encephalitis homozygous for a *DBR1* mutation. (A)** Family pedigree of index patient 1 (P1) homozygous for the *DBR1* I120T/I120T mutation. The segregations of the *DBR1* (I120T) and *MEFV* (M694V, E148Q) variants are indicated. Pathogenic mutations are shown in red for *DBR1* and in blue for *MEFV*. Siblings are labeled from sibling 1 (S1) to S6. **(B)** Estimation of a potential common haplotype surrounding the *DBR1* I120T mutation, as predicted by EstiAge analysis, for P1 and a previously reported patient with HSV-1 BVE. **(C)** MRI fluid–attenuated inversion recovery images taken on day 2 of encephalitis in P1.

We analyzed the 16 exomes for candidate genotypes for the 19 known viral encephalitis–causing genes (*TLR3*, *UNC93B1*, *TRIF*, *TRAF3*, *TBK1*, *IRF3*, *NEMO*, *GTF3A*, *MDA5*, *DOCK2*, *POL3A*, *POL3C*, *IFNAR1*, *STAT1*, *STAT2*, *TYK2*, *SNORA31*, *DBR1*, and *RIPK3*) (Casanova and Abel, 2021; Liu et al., 2023; Zhang et al., 2021), and for another three genes implicated in inborn errors of immunity (IEI) and with functions closely related to those of the known viral encephalitis–causing genes (*IFNAR2*, *IRF9*, and *JAK1*) (Meyts and Casanova, 2021). We searched for rare (minor allele frequency <0.01 in the Genome Aggregation Database and in our in-house whole exome sequencing database containing about 20,000 exomes or genomes) non-synonymous or splicing (affecting essential-splicing or intronic branch-point sites) variants with a combined annotation-dependent depletion score (Kircher et al., 2014) above the mutation significance cutoff (Itan et al., 2016) for the 22 genes, all of which have a gene damage index below 13.83 (Itan et al., 2015). We considered monoallelic and/or biallelic variants in accordance with the known modes of inheritance of the genes concerned. Interestingly, this search revealed that one patient (P1) was homozygous for a missense mutation (I120T) of *DBR1*, which encodes the RNA lariat–debranching enzyme 1 (DBR1), a previously reported genetic etiology of brainstem viral encephalitis (BVE) (Zhang et al., 2018). More specifically, a detailed analysis of the whole-genome sequencing (WGS) data for P1 and his two parents revealed that P1 was homozygous or compound heterozygous for 18 and 1 genes, respectively (Table S1). In addition, P1 was found to be heterozygous for a known familial Mediterranean fever (FMF)–causing pathogenic *MEFV* gene variant, M694V (Tirosh et al., 2021), and another *MEFV* variant, E148Q, previously reported to confer mild genotype modifications (Fig. 1 A). The *DBR1* variant of P1 was previously shown to be pathogenic, underlying HSV-1 BVE (Zhang et al., 2018). An EstiAge analysis of sequencing data for P1 and a previously described patient with HSV-1 BVE showed that they had a haplotype of about 6.61 Mb in common, estimated to have originated from a common ancestor about 21 generations ago (95% confidence interval: 7–85 generations), corresponding to a period of about 567 years (297–2,295 years) (Fig. 1 B). None of the other genes were deemed plausible candidates to explain SARS-CoV-2 encephalitis based on the biochemical nature of the variant, the reported tissue expression of the corresponding gene, or the known function of its products.

P1 is a 14-year-old boy born to nonconsanguineous parents of Syrian origin living in Sweden. P1 and one of his six siblings (S6) had a clinical history of FMF, with recurrent episodes of serositis. The familial segregation of the *DBR1* I120T and *MEFV* M694V variants suggested incomplete penetrance of the two genotypes for the BVE and FMF clinical phenotypes, respectively (Fig. 1 A). In February 2022, P1 presented with a sore throat and fever at a time at which other members of his family had mild COVID-19. 1 wk later, he was admitted to an intensive care unit at Umeå University Hospital, Sweden, for acute dizziness, vomiting, and loss of consciousness. Magnetic resonance imaging (MRI) showed brainstem encephalitis, with lesions in the pons, mesencephalon, and cerebellum, and evidence of an increase in intracranial pressure (Fig. 1 C). At admission, PCR on a nasopharyngeal sample was positive for SARS-CoV-2, but no IgG against SARS-CoV-2 was detected in the blood or cerebral spinal fluid (CSF). SARS-CoV-2 was therefore considered to be the virus responsible for encephalitis (Table S2). Extensive searches for other pathogens in the blood and CSF, and screening for neuronal autoantibodies in the blood yielded negative results (Table S3). P1 was treated with an external ventricular drain, corticosteroids, remdesivir, and IL1 blockade, and has no sequelae 15 mo after the encephalitis episode. Immunological studies 6 mo after the recovery of the patient showed leukocyte subset counts and proliferation to be normal. The activation of T and B lymphocytes, and the functions of neutrophils, phagocytes, and the complement system were also normal (Table S3). P1 tested positive for SARS-CoV-2 IgG in serological tests performed at this time point and presented a robust T cell response to the SARS-CoV-2 spike 1 (S1), spike (S), membrane (M), and nucleocapsid (N) peptides, further confirming the history of SARS-CoV-2 infection in this patient.

**Figure S1.**
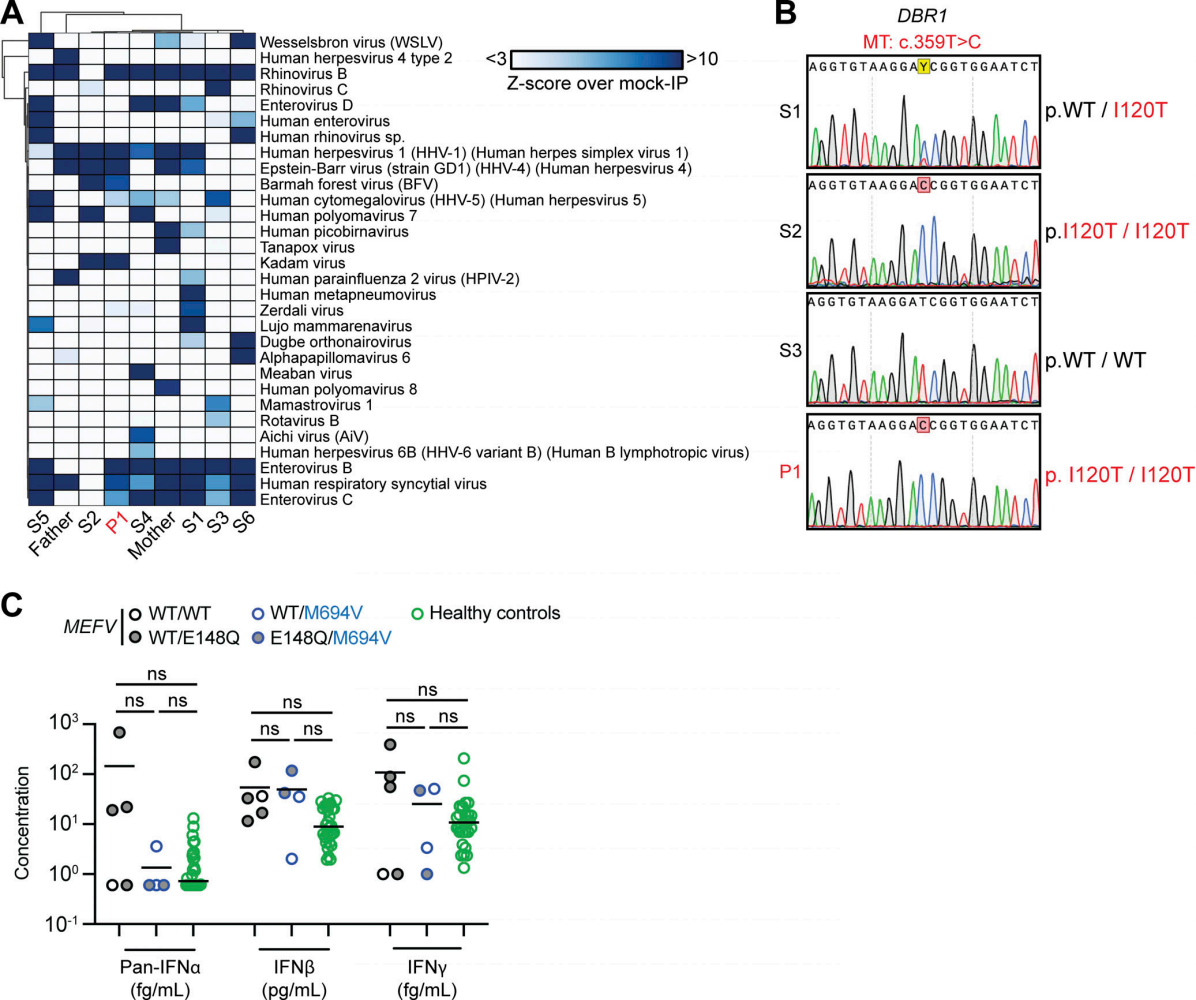
**Homozygosity for the I120T DBR1 variant in a child with isolated SCV-2 BVE. (A)** VirScan test for antibodies against a wide range of viruses in the serum of P1, his siblings, and their parents. Hierarchically clustered (Pearson) heatmap showing PhIP-Seq antibody enrichment (z-score relative to mock immunoprecipitation [IP]) for each of the 30 viruses detected in at least one member of the family. All values are the means of technical duplicates. **(B)** Electropherogram showing the *DBR1* gDNA sequence surrounding the I120T mutation, in P1 and his older siblings (S1 [born 2000], S2 [born 2002], and S3 [born 2004]). **(C)** IFN-α, -β, and -γ levels in the plasma of various members of the family and 30 other healthy controls, as measured by SIMOA digital ELISA. Statistical analysis was conducted with Mann–Whitney *U* tests. ns: not significant.

DBR1 has only one known cellular function, as the only intronic RNA lariat debranching enzyme (Chapman and Boeke, 1991). We previously reported autosomal recessive DBR1 deficiency in five children from three kindreds with brainstem encephalitis due to infection with HSV-1, influenza B virus, or norovirus, with complete clinical penetrance for at least one type of viral encephalitis (Zhang et al., 2018). The I120T mutation was previously found in the homozygous state in two patients with HSV-1 BVE. The I120T variant has been shown to be biochemically deleterious, as it results in abnormally low levels of the corresponding protein and RNA lariat–debranching activity (Zhang et al., 2018). P1 and three of his siblings, aged 6 (S6), 10 (S4), and 20 (S2) years, were found to be homozygous for the I120T variant. None of these three siblings of P1 developed SARS-CoV-2 encephalitis or another severe infection, despite living in the same household as P1 and probably being exposed to the same infectious agents as P1, including viruses (Fig. S1 A). The 6-year-old sibling (S6) was the only sibling with a positive T cell response to SARS-CoV-2 S1 and SMN peptides at the latest follow-up, and this child also had a positive result in the anti-SARS-CoV-2 spike protein serological test (Table S4). The parents were heterozygous and the other three siblings tested (S1, S3, and S5) were heterozygous or WT at the I120 position (Fig. 1 A and Fig. S1 B). These findings are suggestive of autosomal recessive DBR1 deficiency underlying SARS-CoV-2 brainstem encephalitis with incomplete clinical penetrance, consistent with previous reports of IEIs underlying sporadic severe viral diseases (Casanova and Abel, 2021, 2022).

**Figure 2.**
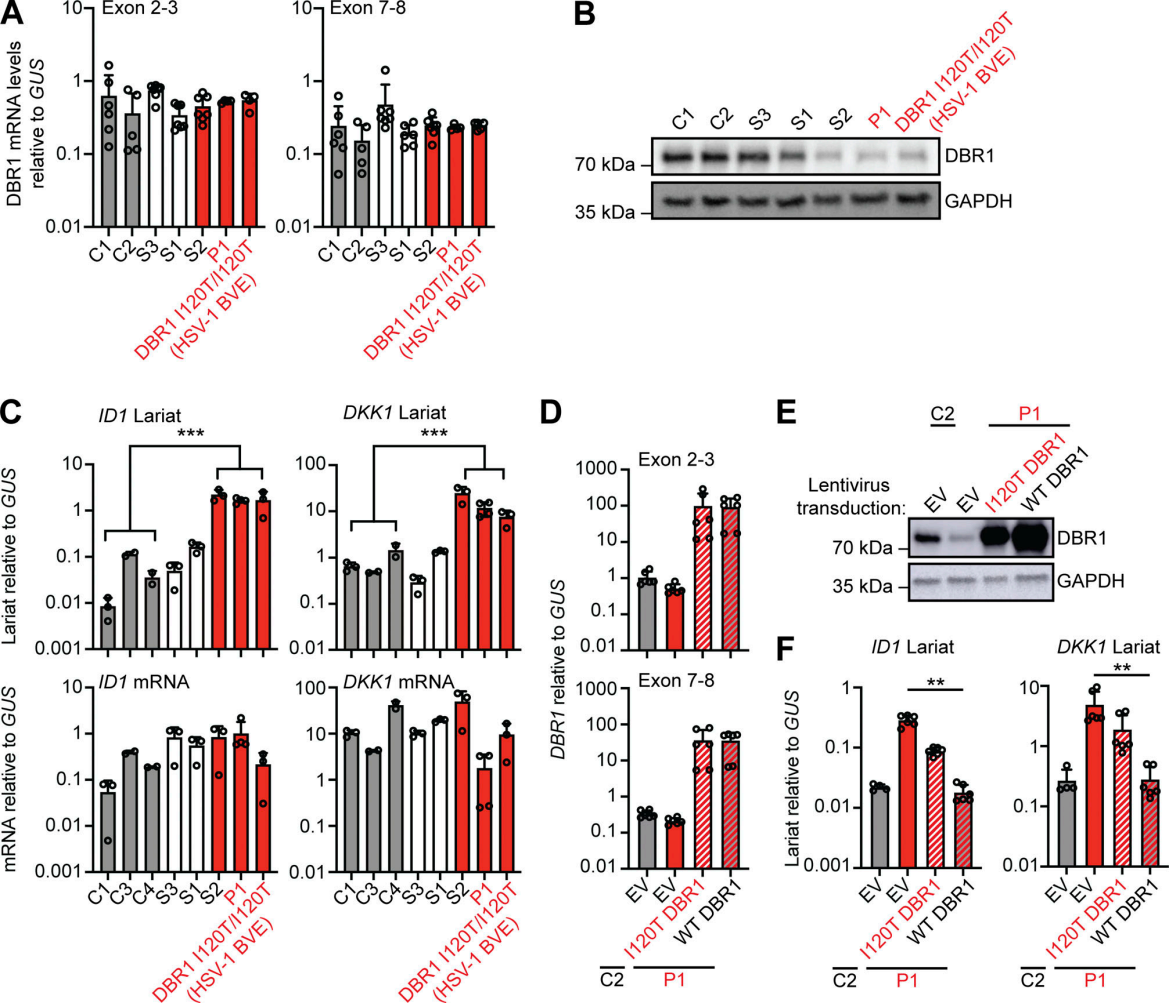
**Intronic RNA lariat levels in patient-derived fibroblasts homozygous for a *DBR1* mutation. (A and B)** DBR1 mRNA levels (A) and DBR1 protein levels (B) in fibroblasts from two healthy controls, a DBR1 WT/WT sibling (S3), a DBR1 WT/I120T sibling (S1), and a DBR1 I120T/I120T sibling (S2) of P1, P1, and a previously reported DBR1 I120T/I120T patient with HSV-1 brainstem encephalitis. **(C)** *ID1* and *DKK1* mRNA and intronic RNA lariat levels, in fibroblasts, as in A and B, as measured by RT-qPCR. Statistical analysis was performed with two-tailed Mann–Whitney *U* test. ***P < 0.001. **(D and E)** DBR1 mRNA levels (D) and DBR1 protein levels (E) in fibroblasts from one healthy control and P1 transduced with empty vector, I120T DBR1, or WT DBR1. **(F)** *ID1* and *DKK1* intronic RNA lariat levels, in fibroblasts, as in D and E, as measured by RT-qPCR. Statistical analysis was performed with two-tailed Mann–Whitney *U* test. **P < 0.01. Data from A, C, D, and F are presented as the means ± SEM from three independent experiments, with two biological replicates for each experiment. Data shown in B and E are representative of three independent experiments. Source data are available for this figure: SourceData F2.

For confirmation of the DBR1 deficiency in P1 at the cellular level, we first studied SV40-transformed fibroblasts (SV40-fibroblasts) as a surrogate cellular model for tissue-resident cells, as in our previous studies (Zhang et al., 2018). SV40-fibroblasts from P1 and one of his siblings (S2) homozygous for DBR1 I120T contain low levels of DBR1 protein and high levels of *DKK1* and *ID1* RNA lariats, like SV40-fibroblasts from one of the previously reported I120T homozygotes (Zhang et al., 2018). This was not the case for the siblings of P1 heterozygous for I120T or WT at this position (Fig. 2, A–C and Fig. S1 B). Exogenous WT *DBR1* expression in SV40-fibroblasts from P1 rescued DBR1 protein levels and decreased *DKK1* and *ID1* RNA lariat levels to values similar to those in healthy control cells (Fig. 2, D–F). A previous study reported abnormally high levels of IFN-γ in the blood of FMF patients even during attack-free periods (Köklü et al., 2005). However, the carriers of the FMF genotype (M694V) from this family had basal circulating IFN-α, -β, and -γ levels similar to those in individuals not carrying this variant (Fig. S1 C). The FMF genotype of this family does not, therefore, appear to affect the penetrance or expressivity of DBR1 deficiency through changes in blood type I or II IFN levels in the context of viral infection. Autosomal recessive DBR1 deficiency may, therefore, underlie brainstem SARS-CoV-2 encephalitis in P1.

**Figure 3.**
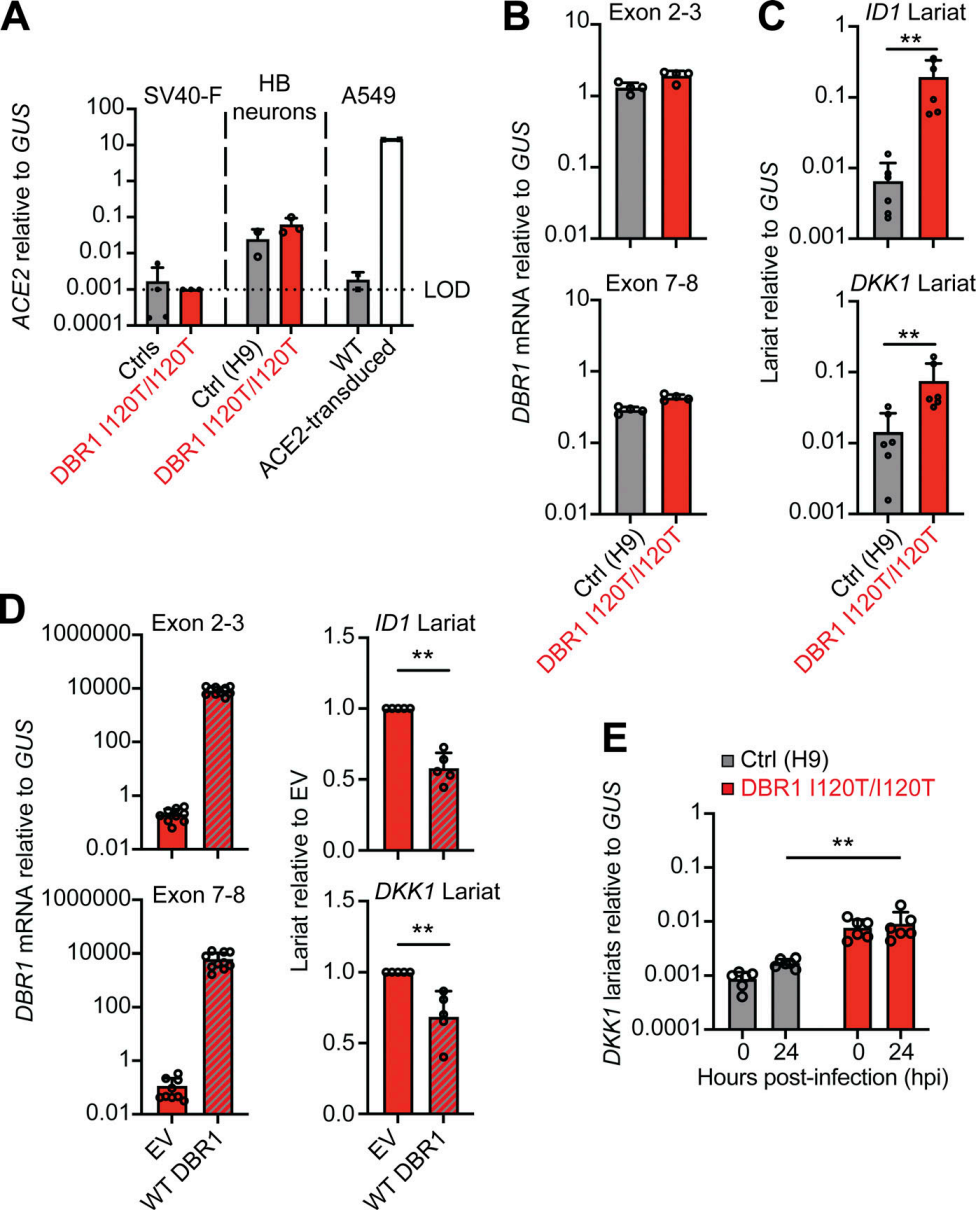
**Intronic RNA lariat levels in hPSC-derived hindbrain neurons homozygous for a *DBR1* mutation. (A)** Angiotensin-converting enzyme 2 (*ACE2*) mRNA levels were determined by RT-qPCR in SV-40 transformed fibroblasts (SV40-F) from healthy controls (C1, C2) and P1, A549 lung carcinoma cells with or without ACE2 transduction, and hPSC-derived hindbrain neurons (HB neurons) from a healthy control (H9) and a previously reported patient with the *DBR1* mutation (DBR1 I120T/I120T). The data shown are the mean ± SEM from two independent experiments, with two technical replicates for each experiment. The limit of detection (LOD) is set as the median of *ACE2* mRNA levels in SV40-F, which does not express ACE2. **(B and C)** *DBR1* mRNA levels (B) and *ID1* and *DKK1* RNA lariat levels (C) in hindbrain neurons derived from healthy control (H9) and DBR1 I120T/I120T patient hPSCs, as measured by RT-qPCR. The data from B and C are presented as means ± SEM from two independent experiments, with two biological replicates for each experiment. **(D)** *DBR1* mRNA levels and *ID1* and *DKK1* RNA lariat levels in hindbrain neurons derived from healthy control and DBR1 I120T/I120T patient hPSCs transduced with empty vector, I120T DBR1, or WT DBR1, as measured by RT-qPCR. The data from D are presented as means ± SEM from two independent experiments, with two biological replicates for each experiment. **(E)** DKK1 lariat RNA levels in hindbrain neurons derived from healthy control and DBR1 I120T/I120T patient hPSCs with or without SARS-CoV-2 infection (MOI 1, 24 hpi). The data shown are the mean ± SEM from three independent experiments, with two technical replicates for each experiment.

**Figure S2.**
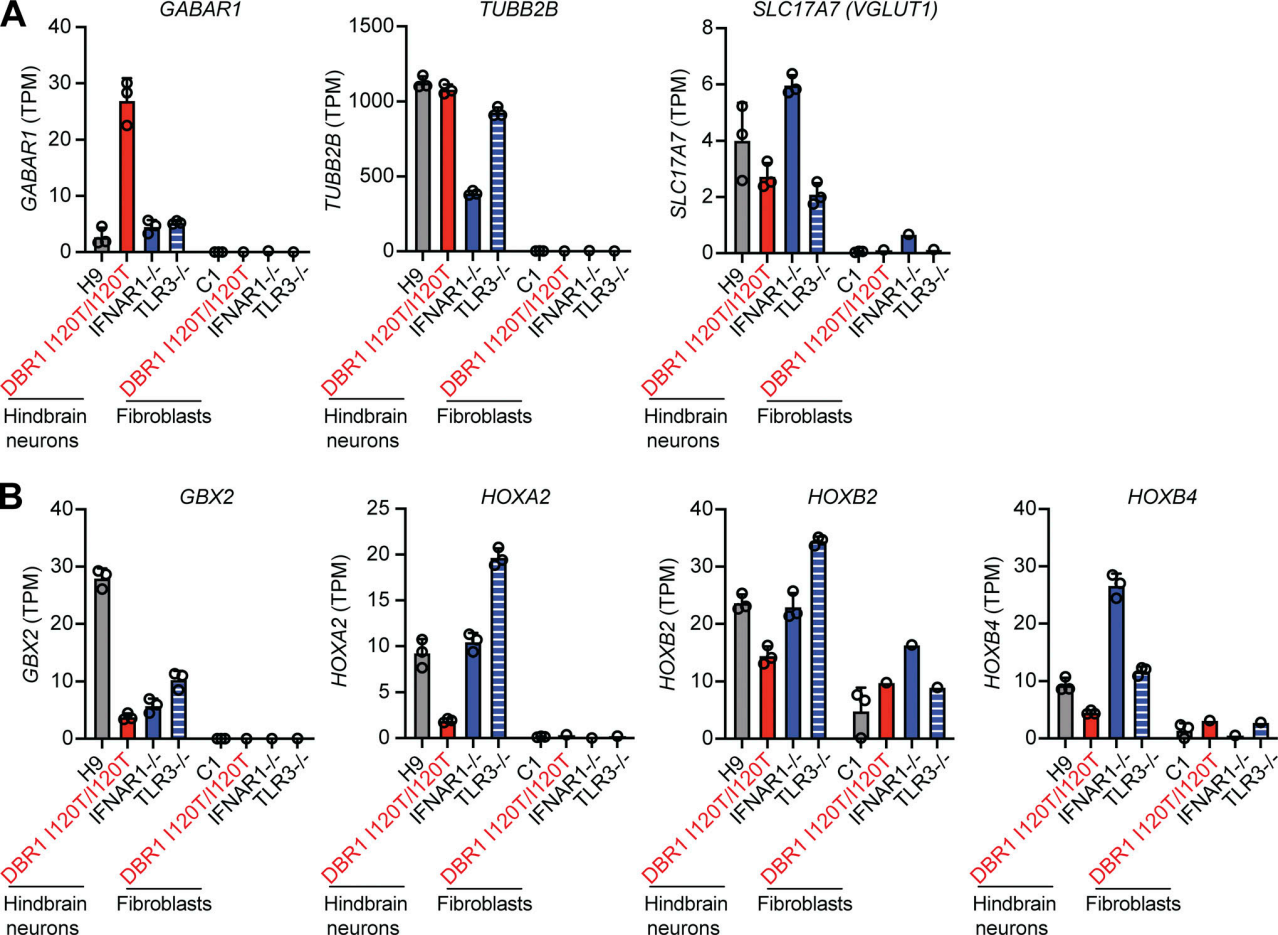
**Characterization of hPSC-derived hindbrain neurons. (A and B)** Abundance of mRNA for the neuronal markers GABAR1, TUBB2B, and SLC17A7 (A), and for the hindbrain neuron–specific markers GBX2, HOXA2, HOXB2, and HOXB4 (B), as assessed by RNAseq, in hPSC-derived hindbrain neurons derived from a healthy control (H9), a DBR1 I120T/I120T patient, an IFNAR1^−/−^ patient, and a TLR3^−/−^ patient. SV40-fibroblasts from a healthy control (C1), a DBR1 I120T/I120T patient, an IFNAR1^−/−^ patient, and a TLR3^−/−^ patient were included as negative controls for the detection of these neuron-specific markers. Triplicates were studied for each sample in A and B.

We previously showed that inherited DBR1 deficiency results in high levels of RNA lariat accumulation, particularly during viral infection, thereby impairing cell-intrinsic antiviral immunity in human fibroblasts (Zhang et al., 2018). The brain-specific mechanisms of DBR1 deficiency impairing cell-intrinsic immunity to viruses remain unclear. DBR1 expression is strongest in the brainstem in humans, consistent with the clinical presentation of the known DBR1-deficient patients, who display selective susceptibility to BVE. We hypothesized that DBR1 deficiency due to homozygosity for the I120T variant would result in uncontrolled SARS-CoV-2 infection in brainstem-resident neuronal cells due to the accumulation of RNA lariats. Hindbrain neurons differentiated from human pluripotent stem cells (hPSC) express angiotensin-converting enzyme 2 (ACE2) and should therefore be permissive for ACE2-mediated SARS-CoV-2 entry (Fig. 3 A). We first assessed the levels of RNA lariats in hindbrain neurons derived from hPSCs from a previously reported DBR1-deficient patient homozygous for the same I120T variant and for similar cells derived from a healthy control (H9). We then assessed the susceptibility to SARS-CoV-2 infection of these cells relative to previously reported patients with forebrain HSV-1 encephalitis and deficiencies of TLR3 or IFNAR1 (TLR3^−/−^, IFNAR1^−/−^) (Bastard et al., 2021b; Guo et al., 2011) and a healthy control (Fig. S2, A and B). Like P1-derived SV40-fibroblasts, *DBR1* I120T/I120T hPSC-derived hindbrain neurons had higher levels of *ID1* and *DKK1* RNA lariats than healthy control neurons (Fig. 3, B and C), and exogenous WT *DBR1* expression decreased the levels of these RNA lariats (Fig. 3 D). SARS-CoV-2 infection also resulted in an increase in *DKK1* lariat levels in both healthy control and DBR1 I120T/I120T hindbrain neurons (Fig. 3 E), as previously reported for the infection of DBR1-mutated dermal fibroblasts with HSV-1 (Zhang et al., 2018). It is, therefore, plausible that SARS-CoV-2 infection is uncontrolled in DBR1-deficient hindbrain neurons due to the accumulation of intronic RNA lariats.

**Figure 4.**
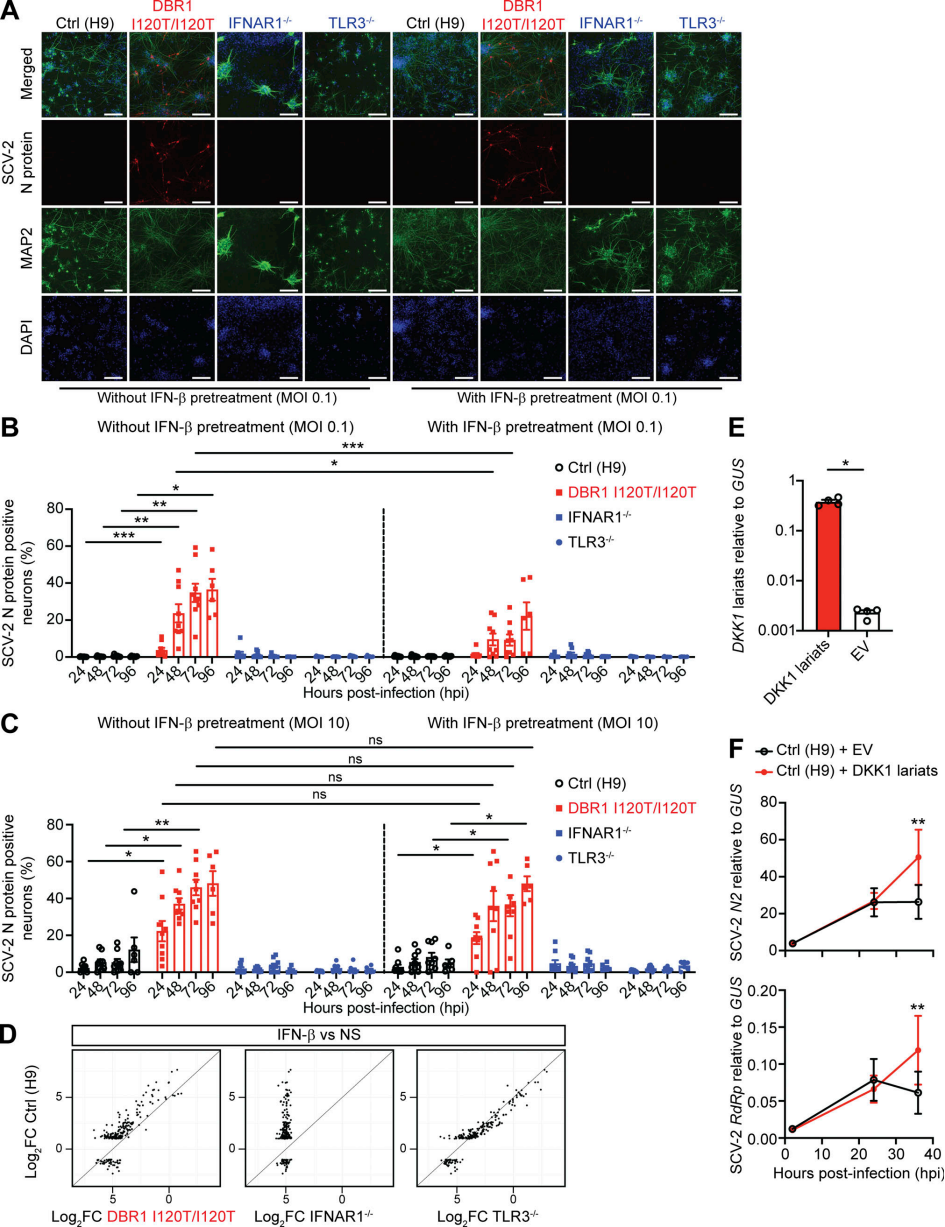
**SARS-CoV-2 infection in hPSC-derived hindbrain neurons. (A)** Representative immunofluorescence images of hPSC-derived hindbrain neurons infected with SARS-CoV-2 (MOI 0.1) at 72 h post-infection (hpi) for a healthy control (H9), a previously reported patient with the *DBR1* mutation (DBR1 I120T/I120T), and patients with complete TLR3 (TLR3^−/−^) or IFNAR1 (IFNAR1^−/−^) deficiency. Cells were stained with antibodies against the SARS-CoV-2 nucleocapsid protein (N, red) and a neuron-specific microtubule-associated protein 2 (MAP2, green). A/T-rich chromosomal DNA was stained with DAPI (blue). Bar: 150 µm. The data shown are representative of three independent experiments. **(B and C)** Percentage of hindbrain neurons (MAP2^+^) positive for the SARS-CoV-2 N protein, at various time points (hpi), with and without IFN-β pretreatment, for cells infected with SARS-CoV-2 at an MOI of 0.1 (B) or 10 (C). The data points are the means ± SEM from three independent experiments with three technical replicates per experiment. Statistical analysis was conducted with Kruskal–Wallis tests, with Dunn’s test for multiple comparisons. *P < 0.05; **P < 0.01; ***P < 0.001. **(D)** Scatterplots of the mean log_2_ fold-changes in RNAseq-quantified gene induction following stimulation with 100 IU/ml of IFN-β for 8 h, in hPSC-derived hindbrain neurons from a healthy control (H9), a previously reported patient with the *DBR1* mutation (DBR1 I120T/I120T), and patients with complete TLR3 (TLR3^−/−^) or IFNAR1 (IFNAR1^−/−^) deficiency. Each point represents a single gene. Genes with an absolute fold-change in expression >2 in response to IFN-β treatment relative to NS samples in the control (Ctrl) group are plotted. **(E and F)** *ID1* and *DKK1* intronic RNA lariat levels (E) and SARS-CoV-2 nucleocapsid 2 (SCV-2 *N2*) and RNA-dependent RNA polymerase (SCV-2 *RdRp*) mRNA levels (F), in hPSC-derived hindbrain neurons from a healthy control (H9) transduced with *DKK1* lariat-expressing lentivirus, as measured by RT-qPCR, after infection with SARS-CoV-2 (MOI 0.1), 2 hpi, 24 hpi and 36 hpi. The data shown are the mean ± SEM from two independent experiments, with two biological replicates for each experiment.

**Figure S3.**
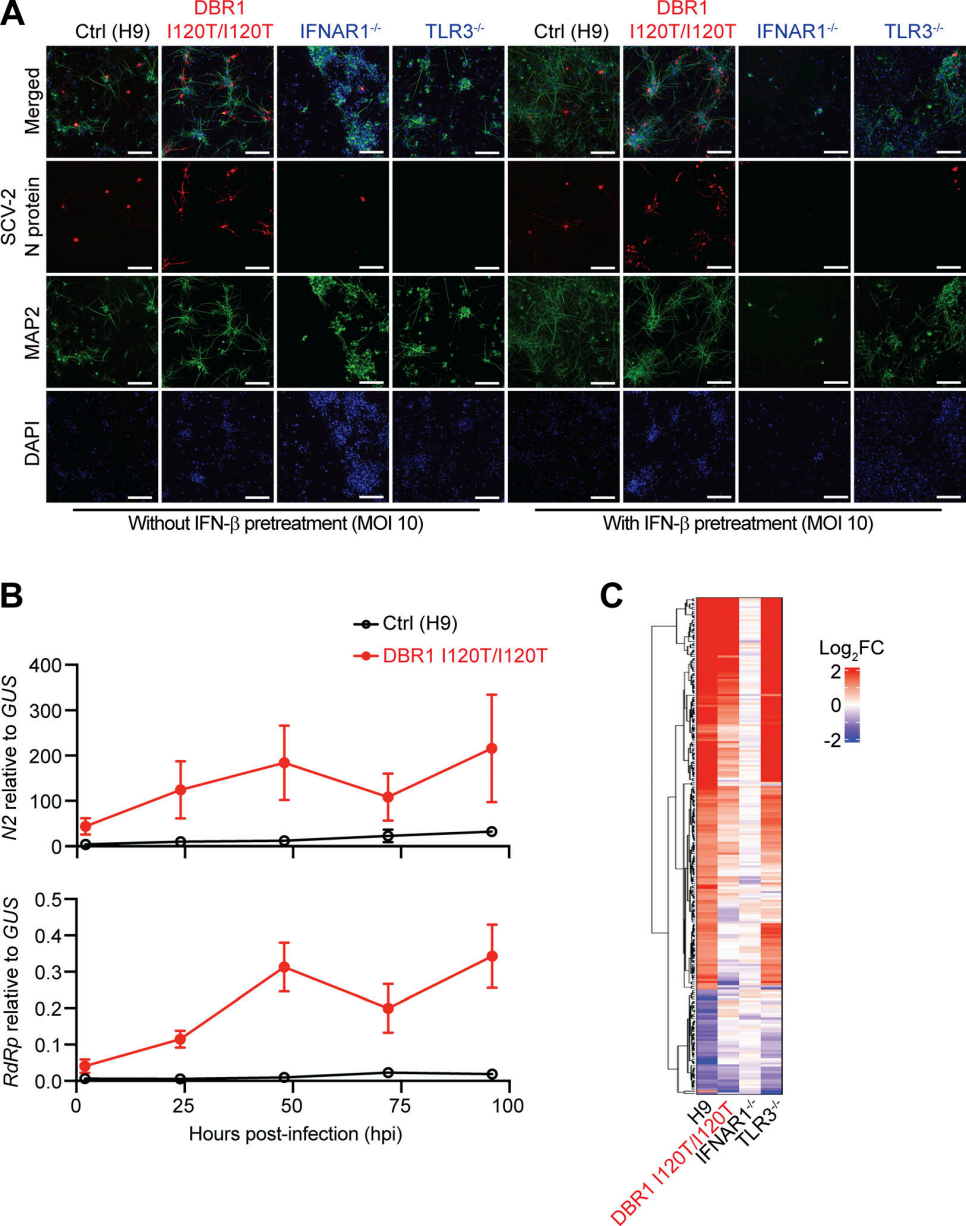
**SARS-CoV-2 infection in hPSC-derived hindbrain neurons with and without IFN-β pretreatment. (A)** Representative immunofluorescence images of hPSC-derived hindbrain neurons infected with SARS-CoV-2 (MOI 10) at 72 hpi, for a healthy control (H9), a previously reported patient with the *DBR1* mutation (DBR1 I120T/I120T), and patients with complete TLR3 (TLR3^−/−^) or IFNAR1 (IFNAR1^−/−^) deficiency. Cells were stained with antibodies against the SARS-CoV-2 nucleocapsid protein (N, red) and a neuron-specific microtubule-associated protein 2 (MAP2, green). A/T-rich chromosomal DNA was stained with DAPI (blue). Bar: 150 µm. Data shown are representative of three independent experiments. **(B)** Quantification of the SARS-CoV-2 nucleocapsid (N2) (upper panel) and the RNA-dependent RNA polymerase (RdRp) (lower panel) by TaqMan real-time qPCR, at 2, 24, 48, 72, and 96 h after SARS-CoV-2 infection (MOI 1). Data are presented as the mean ± SEM and are representative of two independent experiments with biological triplicates in each experiment. **(C)** Heatmaps of RNAseq-quantified gene expression (z-score-scaled DESeq2 vst-normalization) in hPSC-derived hindbrain neurons from a healthy control (H9), a previously reported patient with the *DBR1* mutation (DBR1 I120T/I120T), an IFNAR1^−/−^ patient, and a TLR3^−/−^ H patient, not stimulated (NS) or stimulated with IFN-β for 8 h. Duplicates were studied for each set of conditions and mean gene expression levels were used for subsequent analyses. The heatmap includes genes with a relative fold-change in expression >2 in response to IFN-β treatment relative to NS samples in the control group.

Finally, we studied the susceptibility to SARS-CoV-2 of DBR1-deficient hindbrain neurons relative to cells from TLR3- or IFNAR1-deficient patients with forebrain herpes simplex encephalitis (Bastard et al., 2021b; Guo et al., 2011) and a healthy control. Interestingly, deficiencies of TLR3 or IFNAR1 did not render hindbrain neurons more susceptible to SARS-CoV-2 replication (Fig. 4, A–C; and Fig. S3 A), despite the demonstrated role of such deficiencies in defects of the control of HSV-1 infection in hPSC-derived cortical neurons and of the control of HSV-1, SARS-CoV-2, and other viral infections in human SV40-fibroblasts (Guo et al., 2011; Lim et al., 2019; Zhang et al., 2020; Bastard et al., 2021b). By contrast, *DBR1* I120T/I120T hindbrain neurons displayed markedly higher rates of SARS-CoV-2 replication from 24 to 96 h after infection than hindbrain neurons derived from a healthy control or from TLR3^−/−^ or IFNAR1^−/−^ patients (Fig. 4, A–C; and Fig. S3 B). The TLR3^−/−^ and IFNAR1^−/−^ hindbrain neurons are normally resistant to SARS-CoV-2 infection. Moreover, despite normal cellular responses to IFN-β (Fig. 4 D and Fig. S3 C), prior treatment with IFN-β only partially restricted the replication of the virus in *DBR1* I120T/I120T hindbrain neurons at a low multiplicity of infection (MOI 0.1), with no detectable restriction at a high MOI (MOI 10) (Fig. 4, A–C; and Fig. S3 A). IFN-β treatment also had no detectable effect in control neurons (Fig. 4, A–C; and Fig. S3 A). Importantly, exogenous *DKK1* RNA lariat expression rendered DBR1 WT healthy control hindbrain neurons susceptible to SARS-CoV-2 infection, mimicking DBR1-deficient neurons (Fig. 4, E and F). These findings suggest that type I IFNs are not necessary to control SARS-CoV-2 in hindbrain neurons and that DBR1 is a critical viral restriction factor in these cells.

This study thus identifies inherited DBR1 deficiency as a genetic cause of SARS-CoV-2 brainstem encephalitis. Mechanistically, DBR1 deficiency disrupts intrinsic immunity to SARS-CoV-2 in the human hindbrain, resulting in uncontrolled viral replication and brainstem encephalitis. SARS-CoV-2 is known to be able to invade the central nervous system via the olfactory bulb (Yang et al., 2020; Ziegler et al., 2020), but SARS-CoV-2 encephalitis has generally been considered to be more of an inflammatory condition than a viral condition due to the lack of positive results for SARS-CoV-2 in PCR on the CSF, the observed responsiveness to immunomodulatory or immunosuppressive treatments, such as immunoglobulins or corticosteroids, and the delayed onset relative to infection in some patients (Cho et al., 2023; Ellul et al., 2020; Siow et al., 2021). Our in vitro findings suggest that brain inflammation may stem from uncontrolled SARS-CoV-2 replication, at least in this patient with inherited DBR1 deficiency who developed encephalitis during acute SARS-CoV-2 infection, but we cannot exclude a role for other cellular mechanisms in vivo. It is possible that similar viral replication–related disease mechanisms occurred in other patients with isolated SARS-CoV-2 encephalitis or encephalitis together with a severe infection of the lung or other organs. It is therefore advisable to administer an antiviral treatment, particularly during the early stage of SARS-CoV-2 encephalitis. Future studies should search for mutations of the genes encoding DBR1 and related molecules in other patients with SARS-CoV-2 encephalitis. Our findings also confirm that DBR1 is a gatekeeper of the human brainstem against various viruses, including not only HSV-1, influenza B virus, and norovirus, but also SARS-CoV-2. Patients with DBR1 deficiency should be vaccinated not only against SARS-CoV-2 but probably also against a broader range of viruses; live-attenuated vaccines may be contraindicated and should be avoided. Despite the recessive mode of inheritance of DBR1 deficiency, its penetrance for viral encephalitis appears to be incomplete. It will now be important to decipher the detailed molecular mechanisms by which the accumulation of intronic lariats impairs cell-intrinsic immunity to viruses in the brainstem. Such studies may pave the way for the development of effective preventive or therapeutic measures for patients prone to brainstem encephalitis.

## Materials and methods

### Human subjects

Informed consent was obtained in Sweden, in accordance with local regulations and a protocol for research on human subjects approved by the Swedish Ethical Review Authority (Dnr 2021-06541-01). Experiments were conducted in the United States and France, in accordance with local regulations and with the approval of the institutional review board of the Rockefeller University and the Institut National de la Santé et de la Recherche Médicale, respectively. Approval was obtained from the French Ethics Committee (Comité de Protection des Personnes), the French National Agency for Medicine and Health Product Safety, the Institut National de la Santé et de la Recherche Médicale in Paris, France (protocol no. C10-13), and the Rockefeller University Institutional Review Board in New York, USA (protocol no. JCA-0700).

### WGS

WGS was performed with the Truseq DNA PCR-free protocol (IIIumina) according to the manufacturer’s instructions. Briefly, 1,100 ng genomic DNA was fragmented into fragments of about 350 bp in length with a Covaris E220. Fragmentation was controlled with a Tapestation 4200 (Agilent). The fragments were subjected to end repair and an A-tail and dual-index adaptors (IDT for Illumina; TruSeq DNA UDI) were ligated to the fragments, which were then subjected to double-sided purification to create a narrow fragment-size distribution. Library quantification was performed with the KAPA Library Quantification Kit (Roche). Sequencing was performed on S4 flow cells with a NovaSeq 6000 sequencer (Illumina) in paired-end 150-bp readout mode with the aim of obtaining 400 million read pairs (a mean coverage of about 30×). Demultiplexing was performed with bcl2fastq2 Conversion Software v2.20 (Illumina). The sequences were aligned with the reference human genome sequence (GRCh37) with BWA. Downstream processing was performed with the Genome Analysis Toolkit (GATK), SAM tools, and Picard Tools (https://broadinstitute.github.io/picard/). Variants were called with GATK Unified Genotyper. All calls with a Phred-scaled SNP quality ≤20 were filtered out.

### Pan-viral serology using VirScan

The VirScan methodology employs programmable phage immunoprecipitation-sequencing (PhIP-Seq), where viral peptides are epitomized on the outer surface of the T7 bacteriophage for precise antibody detection, followed by next-generation deep sequencing as described in the previous studies (Schubert et al., 2019; Xu et al., 2015). The extensive VirScan library comprises 481,966 62-amino acid peptides, designed with a 14-amino acid overlap, spanning comprehensively across full-length vertebrate, mosquito-borne, and tick-borne viral genomes. Our approach to phage immunoprecipitation and sequencing incorporated nuanced modification of well-established PhIP-Seq protocols (Mandel-Brehm et al., 2019; O’Donovan et al., 2020; Schubert et al., 2019). Briefly, 1 μl of human sera was incubated with 500 μl of the VirScan library for 12–18 h at 4°C. All samples were run in two technical replicates. Antibody-bound phages were further subjected to two rounds of immunoprecipitation utilizing a mix of protein A and protein G magnetic beads (Thermo Fisher Scientific), followed by elution and sequencing to unveil the unknown antigen(s).

### Western blot

Total cell extracts were prepared from SV40-fibroblasts from patients or healthy controls. Equal amounts of protein from each sample were separated by SDS-PAGE and blotted onto polyvinylidene difluoride membranes (Bio-Rad). The membranes were then probed with an anti-human DBR1 antibody (ProteinTech). They were then stripped and reprobed with an anti-GAPDH antibody (Santa-Cruz) to control for protein loading. Antibody binding was detected by enhanced chemiluminescence (ECL; Amersham-Pharmacia-Biotech) with an Amersham Imager 600 (GE Life Sciences).

### Reverse transcription–quantitative PCR (RT-qPCR)

Total RNA was isolated from the patient’s SV-40 fibroblasts with the Quick-RNA Microprep kit (Zymo Research). We reverse-transcribed the extracted total RNA with random hexamers and the SuperScript III First-Strand Synthesis system (#18080051; Thermo Fisher Scientific). RT-qPCR was performed with Applied Biosystems TaqMan assays with Hs01113902_m1 (spanning DBR1 exons 2–3) and Hs01113907_m1 (exons 7–8) probes for DBR1 and with the β-glucuronidase (#4310888E; GUS) housekeeping gene used for normalization. Results were expressed according to the ΔΔCt method, performed in accordance with the manufacturer’s instructions.

For the quantification of ID1 and DKK1 intron lariats, we reverse-transcribed the extracted total RNA with 0.5 µM branch point-specific reverse primer for ID1 or DKK1 and a reverse primer for β-glucuronidase (GUS) for normalization. The ID1 and DKK1 mRNA transcripts were reverse-transcribed with random hexamers and quantified with primers for ID1 or DKK1 transcripts in the Fast SYBR Green System (#4385616; Thermo Fisher Scientific). The levels of ID1 and DKK1 lariats and transcripts were normalized relative to that of GUS transcripts and calculated according to the ∆CT method. The sequences of the primers used have been reported elsewhere (Zhang et al., 2018).

### SIMOA digital ELISA

Pan-IFNα, IFNγ (duplex), and IFNβ (single-plex) protein concentrations were quantified in SIMOA digital ELISA assays developed as Quanterix Homebrews according to the manufacturer’s instructions. The limit of detection of these assays was 0.8 fg ml^−1^ for IFN-α, 20 fg ml^−1^ for IFN-γ, and 0.2 pg ml^−1^ for IFN-β, considering the dilution factor applied.

### Fibroblast cell culture

Primary fibroblasts were isolated from skin punch biopsy specimens under sterile conditions and were cultured in DMEM (GIBCO BRL; Invitrogen) supplemented with 10% fetal calf serum (FCS) (GIBCO BRL; Invitrogen). Immortalized SV40-transformed fibroblast cell lines (SV40-fibroblasts) were created by the electroporation of about five million cells with 4 mg of a plasmid containing T-antigen DNA. The transfected cells were transferred to two fresh 75-cm^2^ flasks, each containing 12 ml DMEM (GIBCO BRL; Invitrogen) supplemented with 10% FCS (GIBCO BRL; Invitrogen). SV40-fibroblast clones appeared after about 15 days. These clones were cultured and passaged for experimental use.

### Plasmids

The DBR1 (accession #Q9UK59) cDNA was inserted into the pDONOR vector. Site-directed mutagenesis was performed to obtain the mutant I120T DBR1 construct. WT DBR1 and I120T DBR1 constructs were then inserted into the pTRIP vector. To generate DKK1 lariat copGFP-split plasmid, the copGFP fragment was PCR-amplified from the PTY-copGFP plasmid and assembled with DKK1 into a PTY plasmid. DKK1 lariat copGFP constructs were then inserted into the pTRIP vector. All primers used for site-directed mutagenesis or subcloning were generated by SnapGene software (version 7). For lentiviral vector production, envelope plasmid pCMV-VSV-G, packaging plasmid PsPAX2, and transfer plasmid pTRIP were used. Lentivirus was concentrated with a Lenti-X concentrator (Takara Bio). All constructs were sequenced to ensure that no adventitious mutations were generated during the cloning process.

### Patient-specific induced pluripotent stem cell (iPSC) reprogramming, culture, and characterization

Patient-specific iPSCs were obtained by reprogramming the patient’s primary fibroblasts by infection with the nonintegrating CytoTune Sendai viral vector kit (Life Technologies). All reprogrammed cells were karyotyped to ensure that they carried no chromosomal abnormalities. Patient-specific *DBR1* mutations were confirmed by Sanger sequencing of genomic DNA extracted from the iPSC lines. Human iPSC cultures were maintained in Essential 8 medium (#A1517001; Life Technologies) on vitronectin-N (VTN-N, #A14700; Thermo Fisher Scientific)-coated plates. Healthy control hESC line H9 and two experimental control iPSC lines from a TLR3^−/−^ patient and an IFNAR1^−/−^ patient with established deficiencies of the TLR3-type-I IFN circuit (Bastard et al., 2021b; Guo et al., 2011) were used in this study.

### Hindbrain neuron differentiation from hPSCs

The differentiation of hESCs or iPSCs (referred to jointly as hPSCs) into hindbrain neurons was induced by dual-SMAD inhibition (Chambers et al., 2009), with correct anterior-to-posterior patterning achieved by Wnt activation. Briefly, hPSCs were dissociated into a single-cell suspension with Accutase (#AT104; Innovative Cell Technologies) and used to seed Geltrex (#A1413202; Thermo Fisher Scientific)-coated plastic plates at a density of 250,000 cells/cm^2^ in E8 medium supplemented with 10 μM ROCK inhibitor (Y-27632; 10 µM #1254/10; R&D Systems). The cells were then transferred to neural induction medium for 11 days. This medium consisted of E6 medium supplemented with LDN193189 (100 nM #04-0074; Reprocell) and SB431542 (10 μM #1614/50; R&D Systems), with the addition of CHIR99021 (3 μM #4423; Tocris Bioscience) for the first 2 days. After 11 days, this medium was replaced by neural differentiation medium consisting of 1:1 DMEM/F12 and Neurobasal, 1× N2 supplement (#17502-048; Thermo Fisher Scientific), 1× B27 without vitamin A (#12587010; Thermo Fisher Scientific), and 1× penicillin/streptomycin (#15-140-122; Thermo Fisher Scientific). The cells were incubated in this neural differentiation medium for 4 days, and hindbrain neural progenitors were then either cryopreserved in Stem Cellbanker (#11924; Amsbio) or replated onto poly-ornithine/laminin/fibronectin plates at a density of 1.5 × 10^5^ cells/cm^2^ in maturation medium consisting of Neurobasal, medium 1× B27 without vitamin A, 1× penicillin/streptomycin, 2 mM L-glutamine (#25030081; Thermo Fisher Scientific), dibutyryl cAMP (#D0627; Sigma-Aldrich), 10 μM DAPT (#2634; Tocris), 250 μM ascorbic acid (#A4034; Sigma-Aldrich), 10 ng/ml glial cell line–derived neurotrophic factor, and 10 ng/ml brain-derived neurotrophic factor. We added 10 μM Y-27632 at replating. The medium was replaced every 5 days until day 30, when the hindbrain neurons were used for experiments.

### Virus propagation

The SARS-CoV-2 NYC isolate was obtained from the saliva of a deidentified patient on July 28, 2020. The sequence of the virus is publicly available (GenBank OM345241). The virus isolate was initially amplified in Caco-2 cells (passage 1, or P#1 stock). For the generation of P#2 and P#3 working stocks, Caco-2 cells were infected with the P#1 and P#2 viruses, respectively, at a MOI of 0.05 plaque-forming units (PFU)/cell and incubated for 6 and 7 days, respectively, at 37°C. The virus-containing supernatant was then harvested, clarified by centrifugation (3,000 × *g* for 10 min), and filtered through a disposable vacuum filter system with 0.22 mm pores. The P#3 stock used in this study had a titer of 3.4 × 10^6^ PFU/ml, as determined on Vero E6 cells with a 1% methylcellulose overlay, as previously described (Mendoza et al., 2020).

### Quantification of SARS-CoV-2 infection

Hindbrain neuron progenitors were used to seed 96-well plates at a density of 1.5 × 10^5^ cells/cm^2^ and were differentiated into dorsal hindbrain neurons. After 4 wk in culture, the neurons were left untreated or were treated with IFN-β (1,000 IU/ml) for 18 h and were then infected with SARS-CoV-2 for 24, 48, 72, and 96 h. The cells were fixed with 10% neutral-buffered formalin and stained for SARS-CoV-2 nucleocapsid (#GTX135357; GeneTex) and neuron-specific cytoskeletal protein microtubule-associated protein 2 (MAP2, Ab11267; Abcam). Alexa Fluor 647– and Alexa Fluor 488–conjugated secondary antibodies (Invitrogen), respectively, were used for counterstaining. Plates were imaged with the ImageXpress micro XL High-Content Screening System, and data were analyzed with MetaXpress (Molecular Devices).

### Statistical analysis

The data for hindbrain neuron infection with SARS-CoV-2 were obtained from three biological replicates in three independent experiments. For each biological replicate, three technical replicates were performed and averaged for downstream analyses. Statistical analysis was performed with Kruskal–Wallis tests with Dunn’s correction, and the results are indicated in the corresponding figures and legends (ns, not significant; *P < 0.05; **P < 0.01; ***P < 0.001).

### Online supplemental material

Fig. S1 shows detailed VirScan, DBR1 Sanger sequencing, and levels of circulating type I and II IFNs of P1, his family members, and healthy controls enrolled in the study. Fig. S2 shows the characterization of hPSC-derived hindbrain neurons. Fig. S3 shows the evaluation of SARS-CoV-2 infection in hPSC-derived hindbrain neurons. Table S1 shows the homozygous or compound heterozygous rare nonsynonymous or essential splicing variants found in P1’s WGS data. Table S2 shows the viral PCR and viral antibody serological results of P1. Table S3 shows the leukocyte immunological functional tests for the patient. Table S4 shows the viral serological data from P1 and the family members of the patient.

## Supplementary Material

Table S1 shows homozygous or compound heterozygous rare nonsynonymous or essential-splicing variants found in the patient’s WGS data.

Table S2 shows viral PCR and antibodies studies for the patient.

Table S3 shows leukocyte immunological functional tests for the patient.

Table S4 shows viral serological data from the family members.

SourceData F2 contains original blots for Fig. 2.

## Data Availability

All data supporting the findings of this study are available in the published article and its online supplemental material or available from corresponding authors upon reasonable request in accordance with local regulations and ethical approvals related to studies of human subjects. The raw RNA sequencing (RNAseq) data generated from this study are deposited in the NCBI database under the NCBI-SRA project PRJNA1123312.
