# Supplementary material for: SARS-CoV-2 brainstem encephalitis in human inherited DBR1 deficiency
Source: J Exp Med. 2024 Jul 18;221(9):e20231725. doi: 10.1084/jem.20231725 (PMC11256911; doi:10.1084/jem.20231725)
Supplement: Table S1 — shows homozygous or compound heterozygous rare nonsynonymous or essential-splicing variants found in the patient’s WGS data. [file JEM_20231725_TableS1.docx]

**Table S1. Homozygous or compound heterozygous rare nonsynonymous or essential-splicing variants found in the patient’s WGS data**

| **Gene** | **GDI** | **Variation type** | **Change** | **Zygosity** | **MAF (gnomAD)** | **CADD** |
| --- | --- | --- | --- | --- | --- | --- |
| *ARHGEF26* | 8.25 | missense | p.Phe9Ile | hom | 0.00659824 | 23 |
| *CREBBP* | 3.97 | missense | p.Leu551Ile | hom | 0.00474553 | 22.1 |
| *EDA2R* | 1.1 | missense | p.Arg20Gln | hom | 4.57E-05 | 12.52 |
| *ENOX2* | 1.37 | missense | p.Glu517Lys | hom | 4.57E-04 | 23.2 |
| *PLCH2* | 4.82 | missense | p.Asp23Tyr | hom | - | 13.1 |
| *FAM231B* | 1.21 | indel-frameshift | p.Trp72fs | hom | - | 12.85 |
| *FOXO6* | 8.36 | indel-frameshift | p.Gln337fs | hom | - | 23.8 |
| *PGBD2* | 3.98 | missense | p.Ile117Thr | hom | - | 10.95 |
| *DBR1* | 2.47 | missense | p.Ile120Thr | hom | - | 27.3 |
| *AC008914.1^*^* | 0.54 | indel-frameshift | p.Thr19fs | hom | - | 22.4 |
| *EEF1E1^*^* | 0.4 | missense | p.Val71Gly | hom | - | 28.1 |
| *HRCT1^*^* | 11.12 | indel-inframe | p.His105del | hom | - | 10.1 |
| *AL138834.1^*^* | - | essential_splicing | - | hom | - | 19.99 |
| *ZNF417* | 4.69 | indel-frameshift | p.His40fs | hom | - | 22.6 |
| *GPR101* | 11.38 | missense | p.Pro500Arg | hom | - | 12.76 |
| *AVPR2* | 3.35 | missense | p.Ser263Ala | hom | - | 23 |
| *F8* | 4.03 | missense | p.Thr1900Ser | hom | - | 23.5 |
| *PCDH11Y^*^* | - | missense | p.Val906Phe | hom | - | 10.67 |
| *TAS2R31* | 7.37 | missense | p.Leu237Phe | het | 0.00850427 | 21 |
|  |  | missense | p.Arg124Gly | het | 6.37E-05 | 23.1 |

Note: Nonsynonymous or essential-splicing variants (with minor allele frequency [MAF] < 0.01 in gnomAD, CADD > MSC of 95% confidence interval, or CADD > 10 when the MSC of 95% confidence interval of a specific gene is not available---as indicated with an asterisk) were found to be in homozygosity or compound heterozygosity, in 18 and 1 gene(s) (with gene damage index [GDI] < 13.83), respectively, in the WGS data of P1.
