# Supplementary material for: SARS-CoV-2 brainstem encephalitis in human inherited DBR1 deficiency
Source: J Exp Med. 2024 Jul 18;221(9):e20231725. doi: 10.1084/jem.20231725 (PMC11256911; doi:10.1084/jem.20231725)
Supplement: Table S2 — shows viral PCR and antibodies studies for the patient. [file JEM_20231725_TableS2.docx]

**Table S2. Viral PCR and antibodies studies for the patient**

|  | **Serum/plasma** | **CSF** | **NPH** |
| --- | --- | --- | --- |
| Sars-CoV-2 PCR | Negative | Negative | Positive |
| Sars-CoV-2 IgG | Negative |  |  |
| Herpes simplex virus type 1 | IgG Positive  IgM Negative | Negative |  |
| Herpes simplex virus type 2 |  | Negative |  |
| Varizella-zoster virus | IgG Positive  IgM Negative | Negative |  |
| Cytomegalovirus | IgG Positive  IgM Negative |  |  |
| Epstein-Barr virus | IgG Positive  IgM Negative | Negative |  |
| Tick-borne encephalitis | Negative |  |  |
| Adenovirus | Negative |  |  |
| Enterovirus | Negative | Negative |  |
| Toxoplasma | Negative |  |  |
| Tularemia | Negative |  |  |
| Neuronal antibodies |  | Negative |  |
| Extractable nuclear antigen (ENA)-screen | Negative |  |  |
| Antinuclear antibody (ANA-Hep-2-IF) | Not detectable |  |  |
| QuantiFERON | Negative |  |  |
| 16S rRNA |  | Negative |  |

NPH, nasopharyngeal swab.
