# Supplementary material for: SARS-CoV-2 brainstem encephalitis in human inherited DBR1 deficiency
Source: J Exp Med. 2024 Jul 18;221(9):e20231725. doi: 10.1084/jem.20231725 (PMC11256911; doi:10.1084/jem.20231725)
Supplement: Table S3 — shows leukocyte immunological functional tests for the patient. [file JEM_20231725_TableS3.docx]

**Table S3. Leukocyte immunological functional tests for the patient**

| **Leukocyte studies** | **Stimulation/analysis** | **Results** |
| --- | --- | --- |
| Lymphocyte populations (flow cytometry) |  | Normal |
| Flow-cytometric assay of specific cell-mediated immune response in activated whole blood, CD4 and CD8 cells | Unstimulated | Normal |
|  | PHA | Normal |
|  | PWM | Normal |
|  | PPD | Normal |
|  | TT | Normal |
|  | Candida | Normal |
|  | Influenza A | Normal |
|  | CMV | Normal |
|  | HSV | Normal |
|  | VZV | Normal |
| T cell response to SARS-CoV-2 | S1 and SMN peptides | Positive |
| Phagocyte function of neutrophils and monocytes | E. coli | Normal |
|  | PMA | Normal |
|  |  |  |
| Antibody levels in serum, IgA, IgG, IgM |  | Normal |
| Complement function | Classic pathway | Normal |
|  | Alternative pathway | Normal |
|  | Lectin pathway | Normal |

PPD, purified protein derivative; TT, tetanus toxoid; VZV, varicella zoster virus.
