# Supplementary material for: SARS-CoV-2 brainstem encephalitis in human inherited DBR1 deficiency
Source: J Exp Med. 2024 Jul 18;221(9):e20231725. doi: 10.1084/jem.20231725 (PMC11256911; doi:10.1084/jem.20231725)
Supplement: Table S4 — shows viral serological data from the family members. [file JEM_20231725_TableS4.docx]

**Table S4. Viral serological data from the family members**

| **Individual** | **SCV-2 Anti-Nucleocapsid** | **SCV-2 Anti-Spike** | **Anti-HSV-1** | **Anti-HSV-2** |
| --- | --- | --- | --- | --- |
| P1 | ? (0.64) | + (37.4) | + (50.3) | <0.5 |
| Father | ? (0.52) | + (1990) | + (31.2) | <0.5 |
| Mother | - (0.02) | - (< 4.81) | + (>62.2) | <0.5 |
| S1 | - (0.23) | - (13.9) | + (38.8) | <0.5 |
| S2 | ? (0.88) | - (29.3) | + (48.7) | <0.5 |
| S3 | + (2.64) | + (401) | + (6.57) | <0.5 |
| S5 | - (0.13) | + (44.6) | + (32.9) | <0.5 |
| S6 | ? (0.42) | + (578) | - (0.277) | <0.5 |
| **Range:** | + (>= 1.4)  ? (0.4 – 1.4)  - (<0.4) | + (>= 33.8)  - (<33.8) | + (>= 1.1)  ? (0.9 – 1.1)  - (<0.9) | N/A |

Note: Serology testing was performed by LIAISON XL fully automated immunoassay analyzer. S4 was not tested due to insufficient serum volume.
