# Supplementary figures and images for: SARS-CoV-2 brainstem encephalitis in human inherited DBR1 deficiency
Source: J Exp Med. 2024 Jul 18;221(9):e20231725. doi: 10.1084/jem.20231725 (PMC11256911; doi:10.1084/jem.20231725)

2B

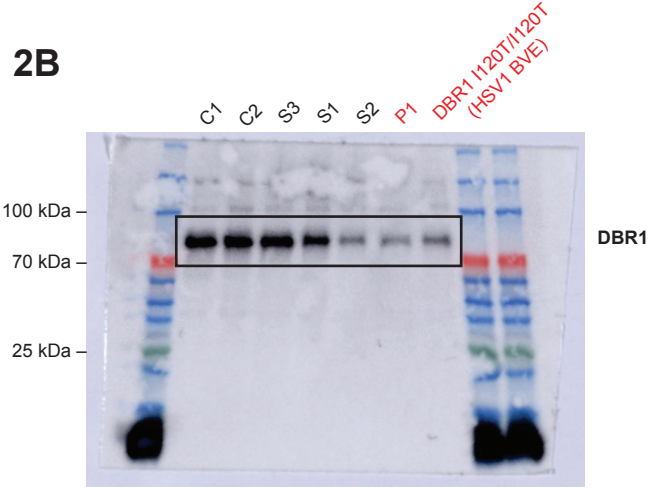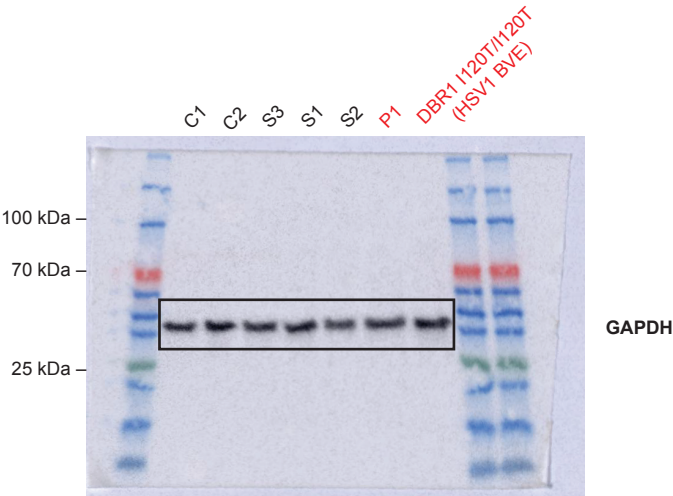

2E

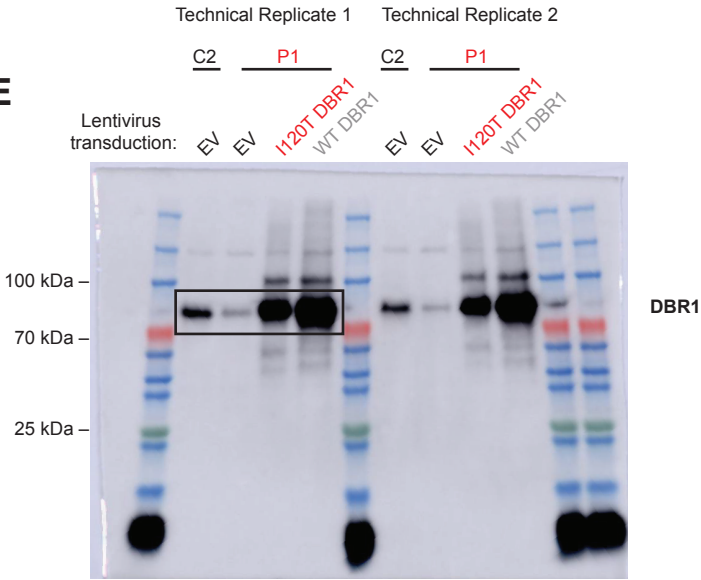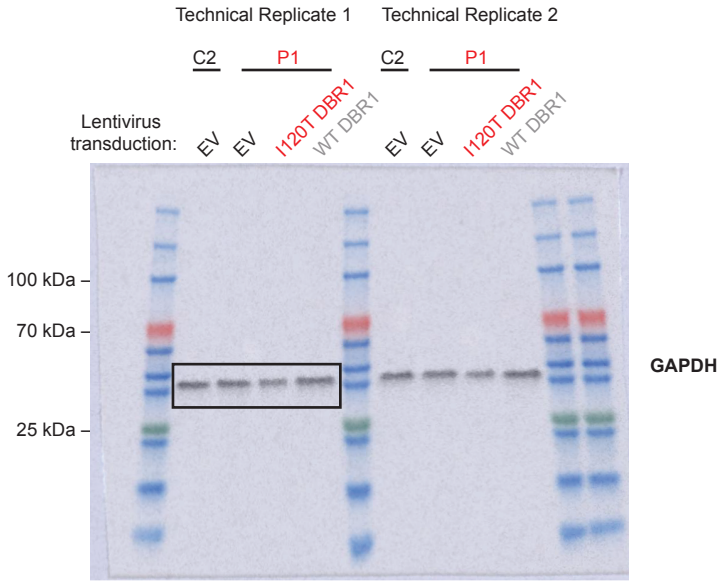

Supplement: SourceData F2 — contains original blots for Fig. 2. [file JEM_20231725_SourceDataF2.pdf]
